# Performance of Motor Sequences in Children at Heightened vs. Low Risk for ASD: A Longitudinal Study from 18 to 36 Months of Age

**DOI:** 10.3389/fpsyg.2016.00724

**Published:** 2016-05-13

**Authors:** Valentina Focaroli, Fabrizio Taffoni, Shelby M. Parsons, Flavio Keller, Jana M. Iverson

**Affiliations:** ^1^Laboratory of Developmental Neuroscience, Università Campus Biomedico di RomaRome, Italy; ^2^Laboratory of Biomedical Robotics and Biomicrosystems, Università Campus Biomedico di RomaRome, Italy; ^3^Department of Psychology, University of PittsburghPittsburgh, PA, USA

**Keywords:** motor development, autism spectrum disorder, fine motor skills, reaching, kinematic data

## Abstract

Recent research shows that motor difficulties are a prominent component of the behavioral profile of autism spectrum disorder (ASD) and are also apparent from early in development in infants who have an older sibling with ASD (High Risk; HR). Delays have been reported for HR infants who do and who do not receive an eventual diagnosis of ASD. A growing body of prospective studies has focused on the emergence of early motor skills primarily during the first year of life. To date, however, relatively little work has examined motor skills in the second and third years. Thus, the present research was designed to investigate motor performance in object transport tasks longitudinally in HR and LR (Low Risk) children between the ages of 18 and 36 months. Participants (15 HR children and 14 LR children) were observed at 18, 24, and 36 months. Children completed two motor tasks, the Ball Task and the Block Task, each of which included two conditions that varied in terms of the precision demands of the goal action. Kinematic data were acquired via two magneto inertial sensors worn on each wrist. In the Block Task, HR children reached more slowly (i.e., mean acceleration was lower) compared to LR children. This finding is in line with growing evidence of early delays in fine motor skills in HR children and suggests that vulnerabilities in motor performance may persist into the preschool years in children at risk for ASD.

## Introduction

Children who have an older sibling with a diagnosis of autism spectrum disorder (ASD) are at heightened risk (HR) for developing ASD and other developmental delays than are children with a typically-developing older sibling and no family history of ASD (Low Risk; LR; Ozonoff et al., 2011; Messinger et al., 2013). Due to the importance of early identification and intervention in achieving positive outcomes for individuals with ASD, there has been a surge of interest in conducting prospective studies of HR infants (see Gliga et al., 2014; Jones et al., 2014, for reviews). One of the most robust findings in this literature is that as a group, HR children, and even those who do not receive an ASD diagnosis, display high inter-individual variability in multiple developmental domains (Zwaigenbaum et al., 2005, 2009). Of importance for the present study is the now widely reported finding of motor delays in HR infants from the first months of life (e.g., Flanagan et al., 2012; Nickel et al., 2013; Libertus et al., 2014). Specifically, at 6 months, HR infants with no subsequent ASD diagnosis have been shown to exhibit less grasping of objects (Kaur et al., 2015) and less bimanual coordination while playing with objects (Bhat et al., 2009) and to spend less time in object mouthing (Koterba and Iverson, 2009; Koterba et al., 2014; Kaur et al., 2015) than their LR peers. They also appear to be delayed in reaching early motor milestones, such as sitting independently (Iverson and Wozniak, 2007; Nickel et al., 2013).

The progressive acquisition of motor skills provides opportunities to acquire and to refine abilities that are relevant in domains beyond motor abilities, such as language and social interaction (Iverson and Goldin-Meadow, 2005; Iverson, 2010). For example, the ability to reach and grasp for an object and to extend it to an interlocutor supports the development of shared attention between infant and caregiver. Karasik et al. (2011) showed that the onset of independent walking influences the quality of infants' social bids. In addition, the ability to manipulate and mouth an object may influence the phonetic characteristics of vocalizations by introducing vocal tract closure and variation in consonant production (Fagan and Iverson, 2007).

A crucial aspect of object manipulation is motor planning. It refers to the capacity to plan the necessary steps to achieve goal directed actions (Gentilucci et al., 1997). Studies of motor planning abilities in children with ASD have suggested some difficulties with planning goal-directed actions globally (i.e., Fabbri-Destro et al., 2009). More generally, deficits or delays in action planning may affect aspects of everyday life. They may also impact social and communicative functioning since the motor system plays a fundamental role in social exchanges. Thus, for example, the ability to plan and produce movements within an appropriate time frame may be crucial for reciprocal social interaction (Zampella and Bennetto, 2013).

To date, the growing literature on motor concerns in HR infants has mainly focused on the first year of life and has examined the attainment of motor milestones, object exploration, and the development of fine and gross motor skills using standardized assessment tools such as the Vineland Adaptive Behavior Scales (Sparrow et al., 2005) and the Mullen Scales of Early Learning Mullen, (1995). In the present study, we extend this line of research by examining the development of the ability to coordinate the motor action sequences needed to transport an object from a starting location to a final target (object transport task) in the second and third years of life. From a neurophysiogical perspective, an action sequence can be defined as a chain of elementary motor acts (e.g., reaching and grasping) that are connected to one another and depend on movement intentions or goals (Fogassi et al., 2005). When an action is performed (e.g., reaching for a block to build a tower), motor acts need to be connected. For instance, when building a tower, children need to lift the hand and reach for a block, shape the fingers to grasp the block, and then place it on top of the target (Sacrey et al., 2014). The final goal of an action sequence guides the relative precision of the actions necessary to accomplish the task (Wilmut et al., 2013). Here we varied the degree of precision required by the final action in order to study potential differences in the performance of goal-directed action sequences.

The present study investigated the development of the ability to execute connected motor acts in HR children who do not go on to receive a diagnosis of ASD and in comparison LR children from 18 to 36 months of age using two different object transport tasks. While previous research on motor development in HR infants has relied on observational methods or administration of standardized assessments, a unique feature of our approach is the combination of behavioral observation with sensor technology specifically developed for use in naturalistic settings that permitted the collection of kinematic data as children performed the tasks. Our aim was to acquire a deeper understanding of the developmental trajectory of motor performance in object transport tasks in HR children and to compare their performance to that of LR peers. Analyses of behavioral and kinematic data allowed us to test for potential differences in task performance as a function of age, condition, and group.

## Materials and methods

### Participants

Fifteen children (8 male) with an older full biological sibling with ASD participated in this research. Children in the HR group were drawn from a larger longitudinal study of the early development of HR infants (e.g., LeBarton and Iverson, 2013). Their families were recruited through a university-based Autism Research Program, parent support organizations, and local agencies and schools serving families of children with ASD. Prior to infant enrollment in the larger study, the Autism Diagnostic Observation Schedule (ADOS; Lord et al., 2000) was administered to all older siblings by a trained clinician to confirm their diagnosis. At 36 months, HR children were seen for final diagnostic assessment and classification by an experienced clinician blind to all previous study data using the ADOS and DSM-IV criteria. All of the HR children scored below the threshold for ASD and did not receive an ASD diagnosis.

The data presented below were collected as part of an ancillary study of motor planning in HR and LR toddlers from 12 to 36 months of age. For purposes of the ancillary study, we recruited a comparison group of 14 LR children (8 male) with an older typically-developing sibling and no family history of ASD (i.e., no first- or second-degree relatives diagnosed with ASD). LR children were recruited via advertisements in local parent magazines, newsletters, neighborhood circulars, pediatricians' offices, daycare and preschool centers, neighborhood email distribution lists, and word of mouth.

All children in both groups were born full-term, from uncomplicated pregnancies and deliveries, and came from English-speaking homes. Although information on family income was unavailable, parental occupations were identified for the purpose of providing a general index of social class. Because many of the mothers were home raising their children, Nakao-Treas occupational prestige scores (Nakao and Treas, 1994) were calculated for fathers' occupations. Groups did not differ statistically in race/ethnicity, maternal or paternal education, or paternal occupational prestige score. Demographic data for the sample are presented in Table 1.

**Table 1:** **Demographic data for HR and LR groups**.

	**HR (*n* = 15)**	**LR (*n* = 14)**
**GENDER**		
Female (%)	7 (47%)	6 (43%)
Male (%)	8 (53%)	8 (57%)
Racial or ethnic minority (%)	0 (0%)	0 (%)
**MATERNAL EDUCATION**		
Graduate or professional school (%)	6 (40%)	5 (36%)
Some college or college degree (%)	7 (47%)	8 (57%)
High school (%)	2 (13%)	1 (7%)
**PATERNAL EDUCATION**		
Graduate or professional schools (%)	6 (40%)	6 (43%)
Some college or college degree (%)	7 (47%)	8 (57%)
High school (%)	2 (13%)	0 (0%)
Mean paternal occupational prestige (SD)	52.91 (15.93)	61.47 (14.71)

### Procedure

Prior to the first ancillary study visit, parents of HR and LR children signed an informed consent form giving permission for their child's participation in the study. All study procedures were approved by the University of Pittsburgh Institutional Review Board. As part of the larger longitudinal study, all HR participants were visited monthly at home between the ages of 5 and 14 months with follow up visits at 18, 24, and 36 months (for further description of the procedures employed in the larger study, see Parladé and Iverson, 2015). For HR children, ancillary study visits generally occurred at a time different from the regularly scheduled visit for the larger study. LR children were seen on or within a few days of the monthly anniversary of their birthday.

**Figure 1:**
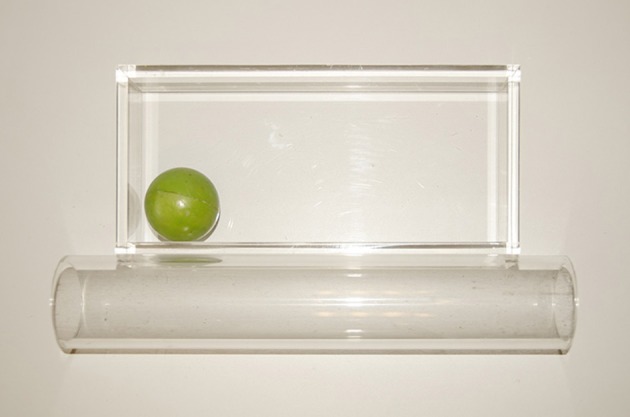
**Stimuli used in the Ball Task: in the Throw condition, the child had to reach for the ball and throw it into the tray; in the Fit condition, the child had to insert the ball into the cylinder**.

All children were seen at home with a primary caregiver for a session lasting ~1 h. Children sat opposite an experimenter, who administered two object transport tasks that increased in level of difficulty. For this reason, tasks were presented in fixed order. Task objects were presented within reaching distance and at the child's midline. Each task involved two conditions differing in the degree of precision required by the goal action. In the Ball Task (adapted from Claxton et al., 2003), children were first administered three trials in which they were asked to throw a small ball (5 cm in diameter) into a transparent plastic tray (30 × 15 × 5 cm). In these *Throw* trials, the goal action (throwing the ball in the tray) does not require precise movement. These were followed by three *Fit* trials, in which children were asked to insert the same ball into a clear plastic tube (6.5 cm in diameter, see Figure 1). In this case, greater precision is required because the center of the ball must be aligned with the axis of the tube for insertion to be successful.

**Figure 2:**
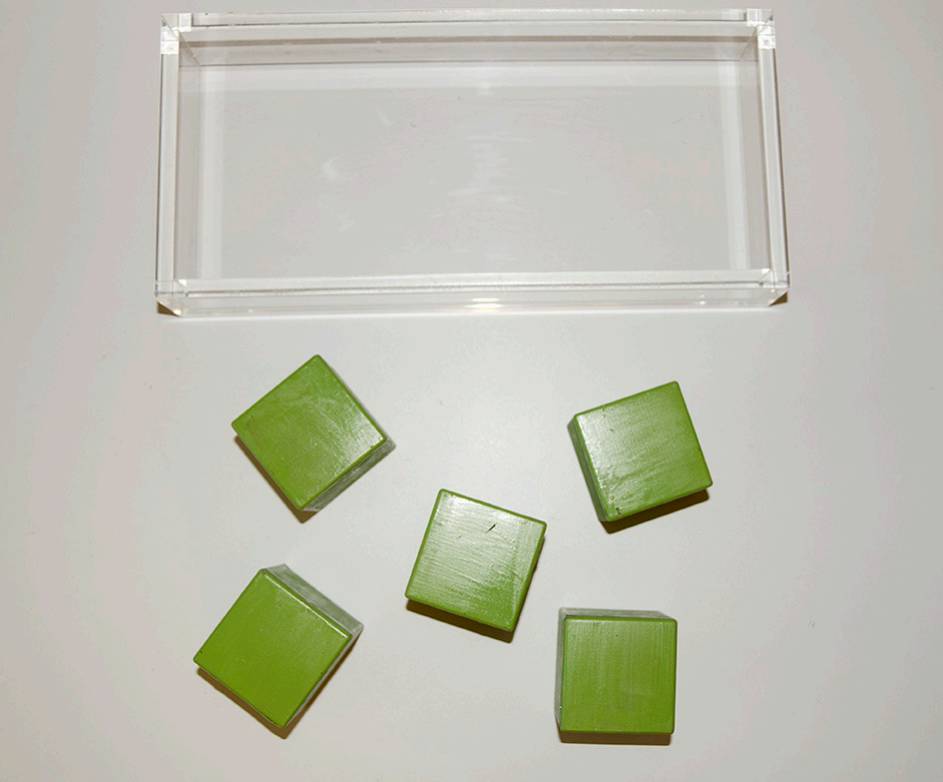
**Stimuli used in the Block Task: in the Throw condition, the child had to reach for the block and throw it into the tray; in the Stack condition, the child had to reach for the block and stack it on a target block to build a tower**.

**Figure 3:**
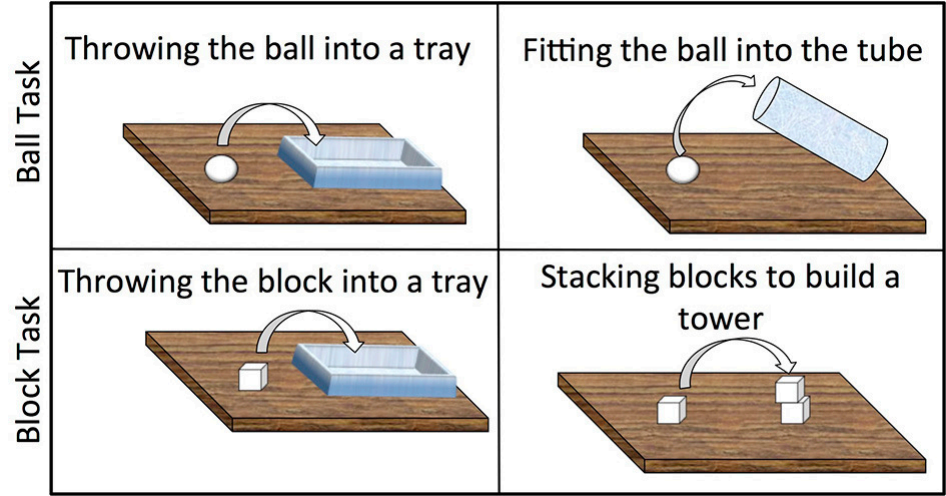
**Schematic representation of the Ball and Block Tasks**. The arrows represent what was to be done with the ball or the block: throw the ball or the block into the tray; fit the ball into the cylinder; stack the block to build a tower.

The second task, the Block Task (adapted from Chen et al., 2010), immediately followed the Ball Task. On the first set of three trials (*Throw* condition), children were asked to throw a block (side 5.5 cm) into a large open container (30 × 15 × 5 cm; see Figure 2). On the next five trials (*Stack* condition), children were asked to place each of five blocks, one at a time, on a target block to build a tower. In these trials, children had to transport the block to the target and then carefully adjust the block to place it successfully on the target block. A schematic representation of the tasks is presented in Figure 3. Sessions were video recorded for later coding.

#### Kinematic data collection

**Figure 4:**
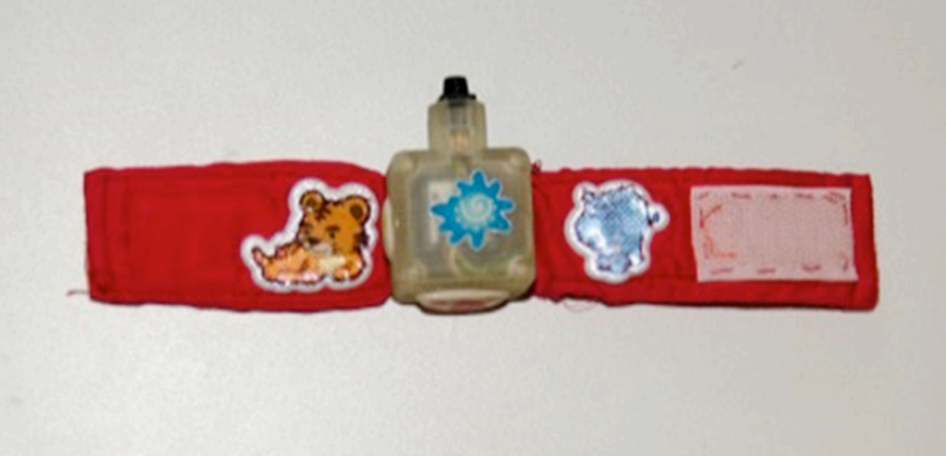
**Sensorized bracelet worn by the children**. We use two identical bracelets, one for the right arm, and one for the left arm, in order to measure children's movement during the execution of the tasks. We used two bracelets since we did not know which arm the child will use to carry out the task.

Kinematic data were collected from the wrists via a magneto-inertial platform consisting of two wrist bracelets (WAMS, Figure 4) instrumented by a 9 axis magneto-inertial sensor (Taffoni et al., 2012). Data were sent to a remote laptop through a Serial-Bluetooth converter (Parani-ESD200, Sena Technologies Inc.). The module allows for a range of 30 m, enabling the monitoring of children in unstructured environments such as the home.

#### Developmental assessments

As part of the larger longitudinal study, the Mullen Scales of Early Learning (MSEL; Mullen, 1995) were administered to all HR infants at 18, 24, and 36 months by a trained researcher. The MSEL provides a measure of general cognitive functioning from 0 to 68 months. It consists of five subscales: Visual Reception, Receptive Language, Expressive Language, Fine Motor, and Gross Motor. Internal consistency ranges from 0.83 to 0.95. The Visual Reception, Fine Motor, Expressive Language, and Receptive Language scores can be used to calculate an overall Early Learning Composite (ELC) *T* score. The MSEL was not administered to LR infants in the ancillary study. MSEL scores for the HR group at 18, 24, and 36 months are presented in Table 2. As is apparent, in general, performance across all five domains fell within the range for the normative sample at each age.

**Table 2:** **Mean Standard (T)^**a**^ Scores (and standard deviations) on the Mullen Scales of Early Learning for HR Toddlers at 18, 24, and 36 months**.

	**18 Months [Mean (SD)]**	**24 Months [Mean (SD)]**	**36 Months [Mean (SD)]**
**MSEL SUBSCALES**			
Gross motor	47.80 (8.91)	42.44 (8.16)	–^b^
Visual reception	49.60 (10.11)	46.38 (6.98)	59.14 (10.15)
Fine motor	53.40 (5.83)	47.23 (7.64)	48.50 (9.51)
Receptive language	36.53 (13.85)	47.69 (12.63)	49.29 (8.21)
Expressive language	42.40 (10.85)	47.85 (8.87)	54.07 (11.39)
Early learning composite^c^	91.47 (13.96)	99.85 (14.12)	105.86 (15.69)

*^a^MSEL subscale T score mean = 50, SD = 10*.

*^b^Because the Gross Motor subscale covers ages birth to 33 months and is not part of the Early Learning Composite, it was not administered at 36 months*.

c *Early Learning Composite T score mean = 100, SD = 15*.

#### Coding and variable creation

Videos were coded by a team of coders naive to children's risk status (HR or LR) using ELAN software.^1^ Prior to commencing coding, all coders were trained to a criterion of 80% agreement on three consecutive training videos. Coding focused specifically on two motor acts: *reaching* and *placement*. *Reaching* began at the first frame in which the child moved the hand from the work surface and ended at the first frame in which the hand contacted the object. *Placement* began from the first frame in which the child lifted the object from the table and ended when the child released it into or on the target. We then calculated the durations of each reach and placement action using these onset and offset times. Interrater reliability was assessed by having a second trained observer independently code a randomly selected 51% of the videos for each task, with the constraint that both groups and all three ages were approximately equally represented in the videos. A tolerance window of 0.1 s was utilized. For the Ball Task, mean intercoder agreement was 0.84 for reach duration and 0.93 for place duration; those for the Block Task were highly comparable (reach duration = 0.83; placement duration = 0.91).

Kinematic data from the WAMS sensors were low pass filtered with a cut-off frequency of 20 Hz to cut noise due to higher frequencies. Filtered data were used to calculate the mean acceleration during reaching movement (Taffoni et al., 2014). The mean acceleration of reaching is a scalar value defined as:

$$\bar{a}=\frac{1}{T}\int_{0}^{T}\left|\vec{a}(t)-R\left(t\right)g_{0}\right|dt$$

*T* is the duration of reaching, $\vec{a}\left(t\right)$ is the accelerometer output at time *t, R(t)* is the orientation matrix describing sensor orientation at time *t* with respect to a fixed reference frame (see Murray et al., 1994), and *g*_0_ is the gravitational acceleration expressed in the same reference frame. The vector difference in the norm operator (|…|) allows subtraction of the gravitational acceleration from the overall acceleration measurement to consider only the acceleration of children's reaching movements. Finally, the integral allowed us to assess the temporal average of the measured acceleration obtaining a scalar metric measuring the performed reaching.

### Statistical analysis

Prior to conducting statistical analyses, we computed a series of *t*-tests to determine whether there were gender differences on any variables. No significant differences emerged, so the analyses reported below were conducted without including gender in the analyses. We utilized random effects regression (STATA 12.1) for our primary analyses. According to Snijders and Bosker (1999), this method accounts for interdependency and structuring of the data and allows the use of multiple data points from the same participant (rather than aggregating all measurements from the same individual and making these values the unit of analysis) while avoiding the problem of pseudoreplication. In addition, the analysis is particularly well suited for analyzing behavioral data that typically have one or more levels of aggregations (Snijders and Bosker, 1999; van de Pol and Wright, 2009). Random effects regression models were computed separately for each task on each of the dependent variables (reaching duration, placement duration, mean acceleration of reaching), with Age (18, 24, 36 months), Condition (*Throw* vs. *Fit*; *Throw* vs. *Stack*), and Group (HR vs. LR) as predictors and with participants as a random factor. The distributions of each dependent variable were checked for normality prior to conducting analyses. Where necessary, appropriate transformations were applied.

## Results

### Ball task

Descriptive statistics for each of the variables from the Ball Task are presented in Table 3. As is apparent, reaching and placement durations varied by age and by condition. Statistical analyses revealed that durations of both actions decreased significantly (reaching duration *z* = 3.23; *p* < 0.01; placement duration *z* = 4.47; *p* < 0.01), while mean acceleration of reaching tended to increase over time (*z* = 4.19; *p* < 0.01). With regard to Condition, placement duration was significantly longer (*z* = −4.66; *p* < 0.01) in the Fit compared to the Throw condition. There was no significant effect of Group on any of the variables examined. Thus, children (regardless of risk status) demonstrated increasing efficiency in executing action sequences over time: reaching movements showed greater acceleration (i.e., higher rate of change in wrist velocity) and were thus shorter in duration, as were placement actions. However, the precision demands of fitting the ball in the tube resulted in longer placement durations compared to the Throw condition.

**Table 3:** **Descriptive statistics from the ball task**.

	**18**			**24**			**36**		
	**Full sample Mean (SD)**	**LR Mean (SD)**	**HR Mean (SD)**	**Full sample Mean (SD)**	**LR Mean (SD)**	**HR Mean (SD)**	**Full sample Mean (SD)**	**LR Mean (SD)**	**HR Mean (SD)**
**THROW**									
Reach duration (s)	0.68 (0.19)	0.72 (0.17)	0.63 (0.19)	0.68 (0.30)	0.71 (0.30)	0.56 (0.30)	0.57 (0.23)	0.51 (0.18)	0.63 (0.26)
Place duration (s)	1.32 (1.52)	1.32 (1.38)	1.31 (1.65)	1.04 (0.56)	0.89 (0.54)	1.16 (0.56)	0.52 (0.19)	0.53 (0.23)	0.50 (0.14)
Mean acc. reaching (m/s^2^)	2.34 (0.79)	2.30 (0.42)	2.38 (1.03)	2.16 (1.33)	2.60 (1.35)	1.61 (1.06)	3.27 (1.50)	3.17 (1.69)	3.38 (1.26)
**FIT**									
Reach duration (s)	0.61 (0.21)	0.62 (0.19)	0.60 (0.23)	0.59 (0.17)	0.60 (0.16)	0.58 (0.17)	0.49 (0.12)	0.47 (0.13)	0.51 (0.12)
Place duration (s)	1.53 (1.07)	1.80 (1.32)	1.30 (0.72)	1.08 (0.82)	0.77 (0.23)	1.35 (1.02)	0.83 (0.34)	0.83 (0.40)	0.82 (0.26)
Mean acc. reaching (m/s^2^)	2.22 (0.71)	2.30 (0.64)	2.11 (0.78)	2.99 (1.41)	2.88 (1.58)	3.17 (1.06)	3.57 (1.62)	3.07 (1.57)	4.10 (1.50)

### Block task

Descriptive statistics from the Block Task are presented in Table 4. These data suggest that overall, as in the Ball Task, there were developmental decreases in reaching and placement durations, while mean reaching acceleration tended to increase. Placement durations were longer in the Stack than in the Throw condition. In addition, mean acceleration of reaching values were higher in the LR compared to the HR group at all three ages. Statistical analyses confirmed these differences. Reaching and placement durations decreased significantly (reaching *z* = 5.38; *p* < 0.01; placement *z* = −5.24; *p* < 0.01) and mean reaching acceleration increased significantly with age (*z* = 2.57; *p* < 0.01). Placement duration was also affected by the precision demands of the goal action, such that durations were longer in the Stack than in the Throw condition (*z* = 7.69; *p* < 0.01). The group difference in mean acceleration of reaching was also significant (*z* = −2.16; *p* = 0.03). Thus, while children in both groups became more efficient in producing action sequences over time, HR children exhibited reduced acceleration in their reaching movements (i.e., rate of change in wrist velocity was slower compared to LR children).

**Table 4:** **Descriptive statistics from the block task**.

	**18**			**24**			**36**		
	**Full sample Mean (SD)**	**LR Mean (SD)**	**HR Mean (SD)**	**Full sample Mean (SD)**	**LR Mean (SD)**	**HR Mean (SD)**	**Full sample Mean (SD)**	**LR Mean (SD)**	**HR Mean (SD)**
**THROW**									
Reach duration (s)	0.60 (0.15)	0.59 (0.13)	0.61 (0.17)	0.55 (0.16)	0.53 (0.14)	0.57 (0.16)	0.45 (0.11)	0.42 (0.11)	0.61 (0.17)
Place duration (s)	1.99 (1.52)	2.13 (1.91)	1.86 (0.98)	1.51 (1.37)	1.46 (1.40)	1.55 (1.34)	0.81 (0.95)	2.13 (1.91)	1.86 (0.98)
Mean acc. reaching (m/s^2^)	3.10 (2.20)	3.84 (2.47)	2.36 (1.57)	2.83 (1.35)	3.03 (1.52)	2.46 (0.88)	3.33 (1.34)	3.84 (2.47)	2.36 (1.57)
**STACK**									
Reach duration (s)	0.61 (0.17)	0.65 (0.20)	0.57 (0.11)	0.58 (0.15)	0.64 (0.13)	0.52 (0.14)	0.49 (0.13)	0.48 (0.12)	0.50 (0.14)
Place duration (s)	2.78 (1.34)	2.68 (1.06)	2.87 (1.54)	2.33 (0.80)	2.50 (0.73)	2.16 (0.83)	1.88 (0.73)	2.10 (0.67)	1.62 (0.71)
Mean acc. reaching (m/s^2^)	2.58 (1.20)	3.12 (1.12)	1.98 (0.96)	2.56 (0.75)	2.47 (0.75)	2.79 (0.71)	3.55 (1.87)	3.75 (1.78)	3.31 (1.95)

Table 5 reports a summary of the statistical effects found in both tasks.

**Table 5:** **Results of the random effects regressions**.

**Dependent variables**	**Independent variables**		
	**Age**	**Condition**	**Group**
**BALL TASK**			
Reach duration	*z* = 3.23^**^	*z* = 2.12	*z* = 0.04
Place duration	*z* = 4.47^**^	*z* = −4.66^**^	*z* = −1.20
Mean acc. reach	*z* = 4.19^**^	*z* = 1.51	*z* = 0.43
**BLOCK TASK**			
Reach duration	*z* = 5.38^**^	*z* = −1.13	*z* = 0.13
Place duration	*z* = −5.24^**^	*z* = 7.69^**^	*z* = −0.23
Mean acc. reach	*z* = 2.57^**^	*z* = -0.27	*z* = −2.16^*^

*Asterisks Mark Significant Effects*.

* *p < 0.05*;

** *p < 0.01*.

## Discussion

### General remarks

In the present study, we investigated the development of the ability to coordinate different action sequences in an object transport task in children at heightened risk for ASD from 18 and to 36 months of age. Our goals were to describe the development of this skill in HR and LR children and to determine whether their performance differed in motor tasks of varying levels of difficulty. Because most previous research on the development of motor abilities in HR children has focused mainly on the first year of life, the present study enhances our understanding of developmental trajectories by examining behavior from 18 months of age. In light of previous findings indicating that the end goal of an action affects how children organize their motor acts (e.g., Claxton et al., 2003), we utilized two object transport tasks that involved conditions differing in the level of precision required by the goal action.

In both tasks, we observed developmental change in the nature of reaching and placement actions, with both becoming temporally shorter. The decrease in reach durations was likely due to the accompanying increase in mean acceleration of the reaching movement, such that with age, children showed more efficient control of the reaching movement. In addition, children's placement actions at all ages were affected by precision manipulations in both tasks. Compared to the imprecise (Throw) condition, placement actions in the precise (Fit; Stack) goal action conditions were longer in duration.

### Group motor differences

Interestingly, differences between the LR and HR groups were only observed in the Block Task, and only in mean acceleration of reaching, with values significantly lower for HR than for LR children. This difference suggests that the Block Task may be more challenging for HR than for LR children. Why might this be the case? One possibility is that the two tasks differ in the degree of difficulty in the precision conditions (Fit vs. Stack). Previous work suggests that when children stack cubes to build a tower, they are guided by internal models of balancing blocks at the geometric center (Karmiloff-Smith and Inhelder, 1975; Krist et al., 2005; Bonawitz et al., 2007). Although the presence of such internal models may guide children's performance, stacking cubes one on top of another to build a tower places additional demands that are not present when fitting a ball in a tube. When building a tower, children must reach for a cube at a fixed location and transport it to the target position, which changes from one cube to the next due to the increasing height of the tower. By contrast, in the Ball Task, the target position is identical from trial to trial.

A second possibility stems from the fact that the two tasks differ in the affordances of the objects on which children acted. In the Ball Task, children manipulated a sphere, which has no privileged affordances. By contrast, in the Block Task children reached for and grasped a cube, which requires more refined manipulation skills. A large body of research has demonstrated that from relatively young ages, infants adjust the aperture and shape of the hand in ways that match characteristics of the target object (e.g., shape, size) during the reaching movement (Lockman et al., 1984; von Hofsten and Fazel-Zandy, 1984; von Hofsten and Rönnqvist, 1988; Ornkloo and von Hofsten, 2007). The lower mean acceleration observed during reaching among HR children may be indicative of difficulty coordinating the approach toward the cube with alterations in hand shape when a target object must be grasped in a particular way in order to be moved from a surface, transported to a new location, and positioned precisely. Such a difficulty may be indicative of vulnerabilities in the prospective control of movement. Some support for this possibility comes from a recent study of reaching in 10-month-old HR and LR infants (Ekberg et al., 2016). In this research, LR and HR infants reached for a ball that was moving down a curvilinear path off an inclined tabletop, and experimenters measured reach latency, or the time between the ball's entry into reaching space and the onset of infants' reaches. Compared to LR infants, who began their reaches about 200 ms *before* the ball entered their reaching space (i.e., they reached predictively), HR infants initiated reaching movements just as the ball entered reaching space.

This interpretation is further supported by studies that have reported grasping delays and difficulties in younger HR infants (e.g., Libertus et al., 2014). Our findings provide a window into the subsequent developmental trajectory of these abilities and suggest that there may be persistent, subtle alterations in fine motor control in HR children. Along these lines, Leonard et al. (2014a) recently reported that HR children who had poor motor skills at 9 months performed poorly on a standardized motor assessment at ages 5–7 years. While these differences may be relatively small and subtle, they may have cascading effects on development in other developmental domains (e.g., language, social; Iverson, 2010; Leonard et al., 2014b). Taken together, our findings and those of prior studies indicate a real need for additional research on grasping in older HR children, and in particular, ways in which modulation of hand aperture and shape for grasping may vary in these children in relation to LR peers. While these are relatively basic components of skilled action, disruptions in any of them may also have significant cascading effects on the organization and planning of movement in daily life.

### Considerations on related cascading effects

There is some evidence that motor skills are related to social, emotional, and communicative functioning. For example, Cummins et al. (2005) showed that children with motor problems demonstrate less skill in emotion recognition. This may impact social interaction abilities since because emotion recognition is foundational for social behaviors such as empathy. There is also some indication of a relation between motor coordination and anxious and depressed behavior in preschoolers. Parents of children with motor difficulties reported higher levels of internalizing behavior problems than did parents of children with typical motor skills (Piek et al., 2008). While these correlational data do not allow us to make inferences about the direction of these relationships, it is clear that motor difficulties can negatively impact children's school performance (e.g., writing, drawing) and participation in games and activities with peers, leaving them at risk for social exclusion and lower self-esteem.

### Limitations and future work

In sum, the data from the present study point to the potential existence of subtle difficulties with fine motor skills in HR children in the second and third years of life. Experimental research has consistently found that children at risk for ASD (i.e., Landa et al., 2013) and children with an ASD diagnosis (i.e., Vernazza-Martin et al., 2005; Ozonoff et al., 2008) experience motor delays that are apparent from early in life. Motor difficulties could be related to neuronal organization and cortical connectivity; they may in fact suggest disrupted fronto-striatal pathways and basal ganglia as well as alterations in cerebellar and brain stem functions (Fournier et al., 2010).

Although these findings add to our understanding of the development of motor skills in HR and LR children in an age range that has received little empirical attention, a note of caution regarding their interpretation is in order. The sample sizes were relatively small, and results clearly merit replication with larger groups of children. In addition, data were collected in a naturalistic setting (children's homes), which precluded the possibility of controlling some aspects of task presentation. Nevertheless, our data highlight the promise of collecting kinematic data in such settings and their potential value in revealing subtle variations in movement organization and quality that cannot be readily observed in video recordings. They also underscore the utility of studying motor behavior in the context of everyday actions that children frequently perform.

## Author contributions

VF provided data coding and elaboration and data analysis. She prepared the manuscript and submitted it after receiving and approving both revisions and comments of all co-authors; FT provided technical support and elaboration of all kinematic data. He also contributed to the manuscript preparation before giving his approval of the present version of the manuscript; SMP provided fundamental support during data acquisition and video coding, she gave precious comments and revisions which were integrated in the last version of the manuscript, that was finally approved. FK contributed to design the experimental protocols and he suggested ideas for the interpretation of specific aspects of results. He also made useful comments for the final discussion of the paper. He then agreed that the present version was ready for the submission. He supervised the Italian group of researchers. JMI gave her fundamental contribute to design the work and the experimental protocols, to the interpretation of data and results. She also provided significant revisions and comments during the different phases of the manuscript elaboration before giving her approval of the final manuscript version. She supervised the US group of researchers and coordinated the whole team.

### Conflict of interest statement

The authors declare that the research was conducted in the absence of any commercial or financial relationships that could be construed as a potential conflict of interest.

## Footnotes

^1^https://tla.mpi.nl/tools/tla-tools/elan/elan-description/
